# Zebrafish as a new model to study effects of periodontal pathogens on cardiovascular diseases

**DOI:** 10.1038/srep36023

**Published:** 2016-10-25

**Authors:** Magdalena Widziolek, Tomasz K. Prajsnar, Simon Tazzyman, Graham P. Stafford, Jan Potempa, Craig Murdoch

**Affiliations:** 1 Department of Microbiology, Faculty of Biochemistry, Biophysics and Biotechnology, Jagiellonian University, Kraków, Poland; 2 The Batson Centre, University of Sheffield, Sheffield, UK; 3 The Krebs Institute, Department of Molecular Biology and Microbiology, University of Sheffield, Sheffield, UK; 4 Department of Infection, Immunity and Cardiovascular Disease, University of Sheffield, Sheffield, UK; 5 School of Clinical Dentistry, University of Sheffield, Sheffield, UK; 6 Department of Oral Immunology and Infectious Diseases, University of Louisville School of Dentistry, Louisville, KY, USA

## Abstract

*Porphyromonas gingivalis (Pg*) is a keystone pathogen in the aetiology of chronic periodontitis. However, recent evidence suggests that the bacterium is also able to enter the bloodstream, interact with host cells and tissues, and ultimately contribute to the pathogenesis of cardiovascular disease (CVD). Here we established a novel zebrafish larvae systemic infection model showing that *Pg* rapidly adheres to and penetrates the zebrafish vascular endothelium causing a dose- and time-dependent mortality with associated development of pericardial oedemas and cardiac damage. The *in vivo* model was then used to probe the role of *Pg* expressed gingipain proteases using systemically delivered gingipain-deficient *Pg* mutants, which displayed significantly reduced zebrafish morbidity and mortality compared to wild-type bacteria. In addition, we used the zebrafish model to show efficacy of a gingipain inhibitor (KYT) on *Pg*-mediated systemic disease, suggesting its potential use therapeutically. Our data reveal the first real-time *in vivo* evidence of intracellular *Pg* within the endothelium of an infection model and establishes that gingipains are crucially linked to systemic disease and potentially contribute to CVD.

*Porphyromonas gingivalis (Pg)* is a Gram-negative anaerobe considered a keystone pathogen in the development of severe periodontitis^1^; a chronic inflammatory disease of the gingiva and tooth supporting structures that, if untreated, leads to tooth loss due to bone resorption and destruction of surrounding soft tissues^2^. *Pg* produces several virulence factors that enable the microbe to colonize the host and evade the immune system^3^. The most predominant of these virulence factors are cysteine proteases termed gingipains that are responsible for the majority of the bacterial proteolytic activity^4^. Gingipains consist of the arginine-specific (RgpA, RgpB) and lysine-specific (Kgp) endopeptidases encoded by *rgpA, rgpB* and *kgp* genes respectively. As well as degrading host proteins to provide nutrients for growth, gingipain activity is also crucial for bacterial adhesion, invasion, host cell death and subversion of the host immune response (reviewed by Guo *et al*. and Hajishengallis)^5^,^6^.

Studies show that *Pg* frequently enters the bloodstream at oral sites through bleeding with tooth brushing or during dental procedures causing a transient bacteremia^7^. Considering the growing number of reports of *Pg* systemic dissemination, it is not surprising that periodontitis has been shown to affect the general health status and there is increasing evidence of its association with systemic inflammation^6^ as well as systemic conditions such as cardiovascular disease^8^, atherosclerosis^9^,^10^ and diabetes^11^. However, to date, little is known about how *Pg* interacts with the vasculature and which virulence factors are important for systemic pathogenesis.

Murine models have been predominantly used to study *Pg* pathogenicity, either in the oral cavity^12^ or systemically^13^,^14^. However, these models are expensive and provide limited data on molecular mechanism, so recently other *in vivo* systems have been developed using lower order organisms. Zebrafish (*Danio rerio)* have been used as an alternative model to study human diseases as they provide physiological and anatomical systems similar to higher organisms but remain affordable. They are easier to genetically manipulate and so generation of knock-out mutants or fluorescently-tagged cell types is more accessible^15^,^16^. Recently, zebrafish larvae have been used as a model system to study human infectious diseases and virulence of bacterial pathogens such as *Enterococcus faecalis*^17^*, Salmonella typhimurium*^18^*, Psudomonas aeruginosa*^19^ and *Staphylococcus aureus*^20^. One of the main advantages for using zebrafish to study infectious diseases is the possibility of non-invasive real-time imaging *in vivo* at the cellular level. Since larval zebrafish are transparent the fate of fluorescently-labelled bacteria after injection into zebrafish larvae can be monitored and their co-localization with fluorescently-tagged host cells examined, making these models an attractive alternative to other vertebrate systems where disease development cannot be visualized^17^,^21^.

In this study we used a zebrafish larvae infection model to study the mechanisms of *Pg* systemic pathogenicity. Using gingipain knock-out mutants and protease inhibitors we identify, for the first time, the crucial role that these proteases play during systemic infection, and show the ability of these bacteria to cross the vasculature and disseminate into surrounding tissues *in vivo*. Data presented here demonstrate that the zebrafish model is suitable to study both the host response to *Pg* infection as well as bacterial virulence and provides a powerful optically and genetically accessible tool to study the systemic pathogenicity of oral bacteria.

## Results

### *P. gingivalis* is pathogenic for zebrafish larvae in a systemic infection model

**Figure 1:**
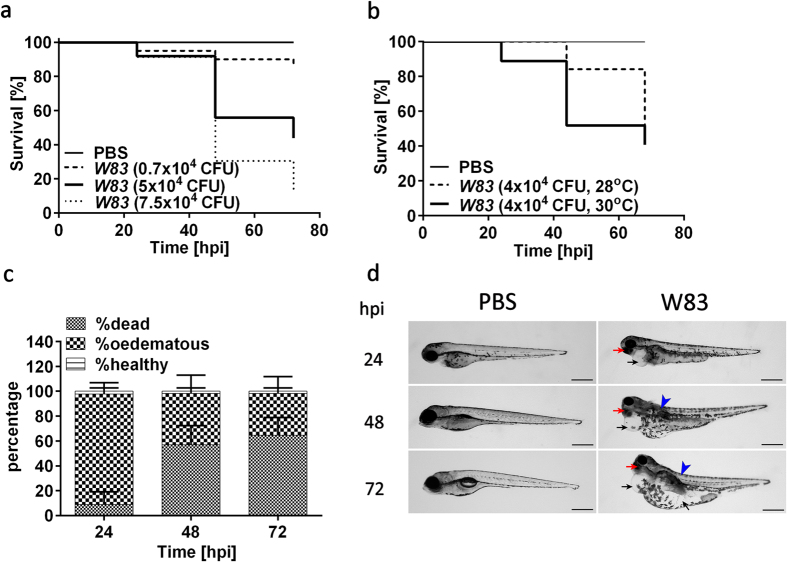
Systemic infection of zebrafish larvae with *Pg* W83 leads to fish mortality and morbidity in a dose- and temperature-dependent manner. Kaplan-Meier survival plots of zebrafish larvae infected with increasing doses of *Pg W83* strain at 30 °C (**a**) or temperature-dependent survival of zebrafish larvae infected with 4 × 10^4^ CFU W83 strain (**b**). At least three individual experiments were performed (n ≥ 20 larvae per group in each experiment). Comparisons between survival curves were made using the log rank test. Levels of dead, oedematous or healthy *Pg W83*-infected larvae for up to 72 h upon injection with 5 × 10^4 ^CFU *Pg W83* (**c**). Development of the oedema disease phenotype in zebrafish larvae infected with *Pg* W83 or PBS-injected controls over 72 h. Black arrows indicate pericardiac and yolk sac oedemas, red arrows indicate eye abnormalities and blue arrowhead indicates spine curving in *W83*-infected larvae. At least three individual experiments were performed (n ≥ 20 larvae per group in each experiment). Scale bars = 500 in (**d**).

**Figure 2:**
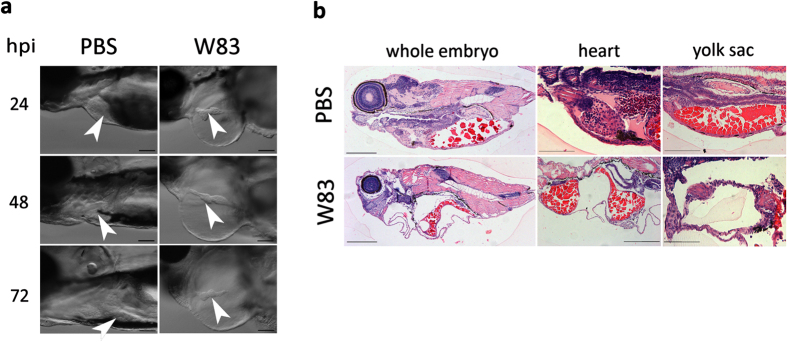
Effect of wild-type *Pg W83* on zebrafish larvae tissue structure. Lateral view of impaired, elongated heart morphology of *Pg W83*-infected larvae; white arrowhead indicates heart. Heart chambers were distant and much smaller in comparison to PBS-injected control larvae (**a**). Sagittal histological sections of H&E stained 48 hpi *Pg W83*-infected larvae revealing advanced tissue damage in the cranial and cardiac regions along with yolk sac oedemas (**b**). In all figures larvae were infected with 5 × 10^4 ^CFU *Pg* at 30 hpf *W83* or PBS as a control. At least three individual experiments were performed and images are representative of at least n = 5 larvae per group in each experiment. Scale bars = 100 μm in (**a**) and 200 μm in (**b**).

To determine whether *Pg W83* strain is virulent in a systemic infection model, zebrafish larvae were injected at 30 hours post-fertilization (hpf) and survival monitored up to 72 hours post-infection (hpi). We observed a dose- and temperature-dependent increase in mortality that was significantly (p < 0.05) different to PBS-injected controls at 48 and 72 hpi (Fig. 1a,b). A LD_50_ was established when 5 × 10^4 ^CFU *Pg W83* were injected into zebrafish for 48 hpi at 30 °C and so these conditions were used throughout the remainder of the study. We noted that zebrafish, remaining viable after infection with *W83*, presented with visible signs of systemic disease that increased with severity overtime compared to controls. In particular, zebrafish displayed severe pericardial and yolk sac oedemas as early as 24 hpi along with eye abnormalities and lordtic spine curving (Fig. 1c,d). In addition, *W83*-infected embryos developed marked cardiac injury as early as 24 hpi. The atrium and ventricle were separated forming thin, elongated chambers within an oedemic appendage and the cardiac tissue displayed extensive damage upon histological analysis (Fig. 2a). Further tissue damage was apparent within the yolk sac and cranial regions that displayed evidence of tissue necrosis (Fig. 2b).

### *P. gingivalis W83* disseminates rapidly in zebrafish larvae, infecting endothelial cells and crossing the vasculature to invade surrounding tissues

**Figure 3:**
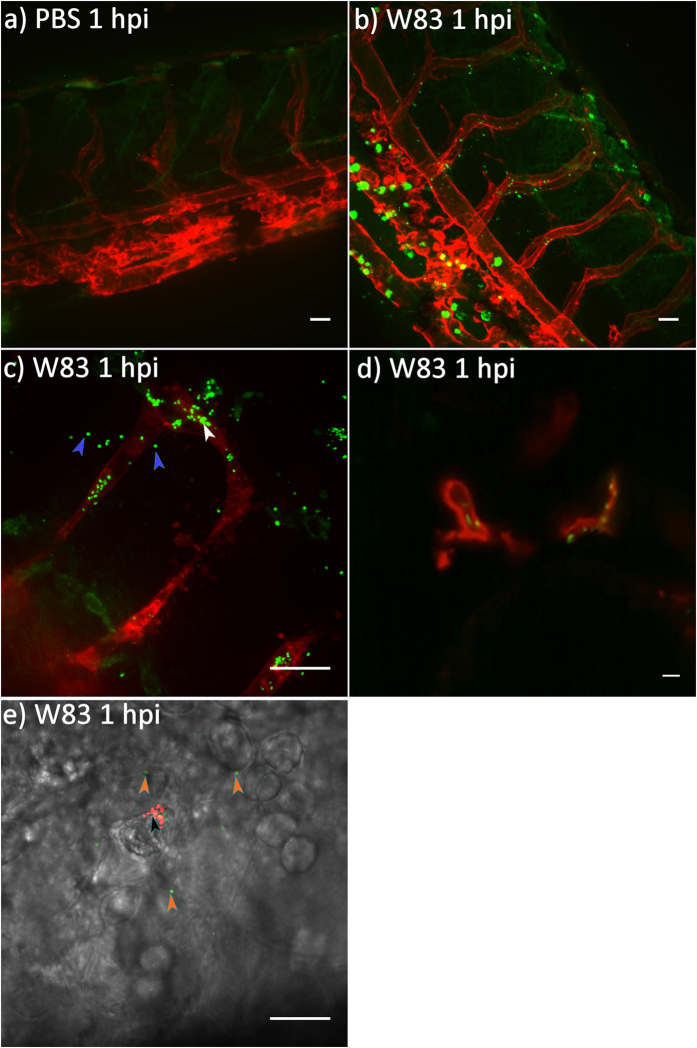
*Pg W83* disseminates rapidly in zebrafish larvae and is able to cross the vascular barrier to invade surrounding tissues. Confocal images of *kdrl:memRFP* transgenic zebrafish larvae at 1 hpi following systemic infection with PBS control (**a**) or fluorescein-labelled 5 × 10^4 ^CFU *Pg W83* (**b**,**c**). Fluorescent *Pg W83* was co-localised to the red-labelled endothelium (white arrowhead in **c**) and cross the vasculature into the tissue (blue arrowheads in **c**). Light sheet image of fluorescein-labelled *Pg* W83 adherent to the heat tissues of zebrafish larvae (**d**). *Pg* are rapidly phagocytosed; high-power image of phagocytosed *Pg* within a phagocyte (red, black arrowhead) and non-phagocytosed *Pg* (green, orange arrowhead) (**e**). Scale bar = 20 μm. Images are representative of two independent experiments showing similar findings.

We next performed real-time, confocal *in vivo* imaging of *kdrl:memRFP* transgenic zebrafish embryos (red-labelled endothelial cells) infected systemically with fluorescently labelled *Pg W83* to determine its cellular targets and if the bacterium could traverse the endothelium. *Pg* was disseminated rapidly in zebrafish and was found associated and co-localised with the inter-segmental endothelial cells at 1 hpi (Fig. 3) providing evidence of adhesion to the endothelium and intracellular localization *in vivo*. In addition, *Pg* was observed outside the red-labelled endothelial cells indicating that bacteria had bound to and crossed the vasculature before entering into the surrounding tissue (Fig. 3C). As well as the inter-segmental vessels we observed *Pg* localization in other highly vascularized tissues including the eye, tail and yolk sac at 1 and 24 hpi (Supplementary Fig. 1). We also observed strong *Pg* adhesion to cardiac tissues where bacteria were not dislodged from tissue despite the intracardiac blood flow (Fig. 3D and Supplementary Movie 1). To visualize the innate immune response to the systemic infection we dual-labelled *Pg* with the pH sensitive dye, pHrodo (non-fluorescent until it is acidified within phagosomes where upon it fluoresces red) and fluorescein to visualize phagocytosed bacteria. After 1 hpi phagocytes had bound and ingested *Pg* and many bacteria were localized within phagosomes (Fig. 3E). Phagocytosis was observed in all tissues examined at this time point and was increased at 24 hpi which was associated with a concurrent decrease in bacterial load (Supplementary Fig. 1).

### *W83* is the most virulent strain of *P. gingivalis* in the zebrafish larvae infection model

**Figure 4:**
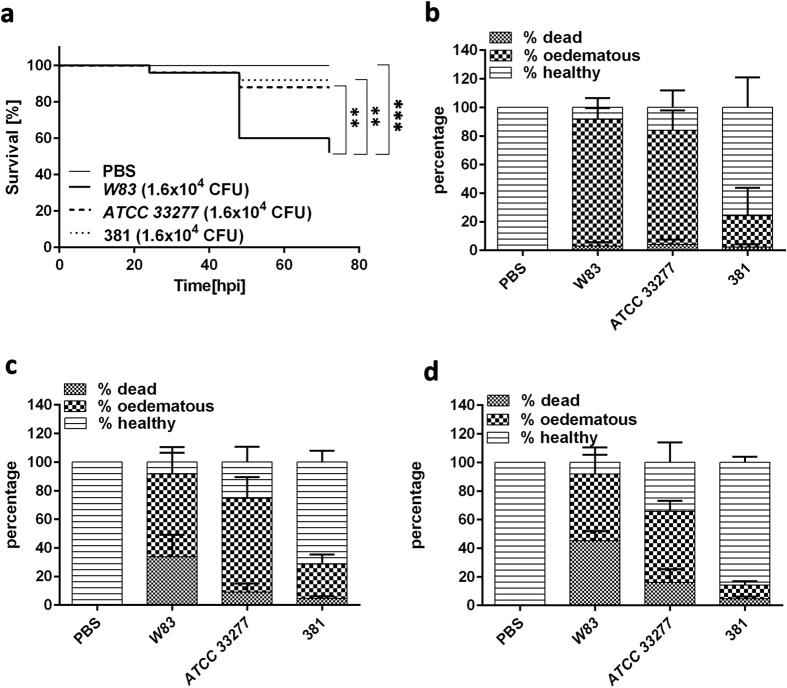
Virulence of *Pg* is strain-specific in the zebrafish larvae infection model. Kaplan-Meier survival plots of zebrafish larvae infected with 1.6 × 10^4 ^CFU of W83, ATCC33277 or FDC 381 strains (**a**). Percentage of dead, oedematous or healthy *Pg W83, ATCC33277* or FDC 381-infected larvae after 24, 48 and 72 hpi respectively (**b**–**d**). Larvae were injected with different strains of *Pg* at 1.6 × 10^4 ^CFU. At least three independent experiments were performed (n ≥ 20 larvae per group in each experiment). **p ≤ 0.01, ***p ≤ 0.001. Comparisons between survival curves were made using the log rank test. Differences between groups displaying oedema were measured using One-way ANOVA after normality was assured.

Previous studies have shown that strains of *Pg* isolated from patients with periodontitis exhibit different levels of pathogenicity in both *in vitro* and *in vivo* models^22^,^23^. We selected three extensively studied strains of *Pg (W83, ATCC 33277* and FDC 381) and compared their virulence in the zebrafish larvae systemic infection model. Kaplan-Meier plots show that strain *W83* was the most virulent in terms of mortality causing significantly more death (p < 0.01) than *ATCC33277* and FDC 381 by 72 hpi (Fig. 4a). In contrast, the virulence of *ATCC33277* and 381 were not significantly different from each other, causing less than 20% mortality of infected larvae by 72 hpi (Fig. 4a). This inter-strain variability was also observed for the development of disease symptoms in infected embryos where infection with *W83* rapidly led to oedemas in the vast majority of infected larvae at 24 hpi with an associated mortality rate of more than 50% of larvae by 72 hpi (Fig. 4b–d). Similarly, 80 ± 14% of *ATCC33277* infected larvae had developed oedemas at 24 hpi but the effect appeared to be less progressive than for *W83* as only 16 ± 9% of larvae were killed by 72 hpi (Fig. 4b–d). *Pg* strain FDC 381 was the least virulent in terms of inducing both systemic disease and mortality. Indeed, some of the larvae displaying oedema were able to recover from this condition by 72 hpi (Fig 4b–d).

### Mortality of *P. gingivalis*-infected zebrafish larvae depends on the viability of the pathogen and its active cell surface components

**Figure 5:**
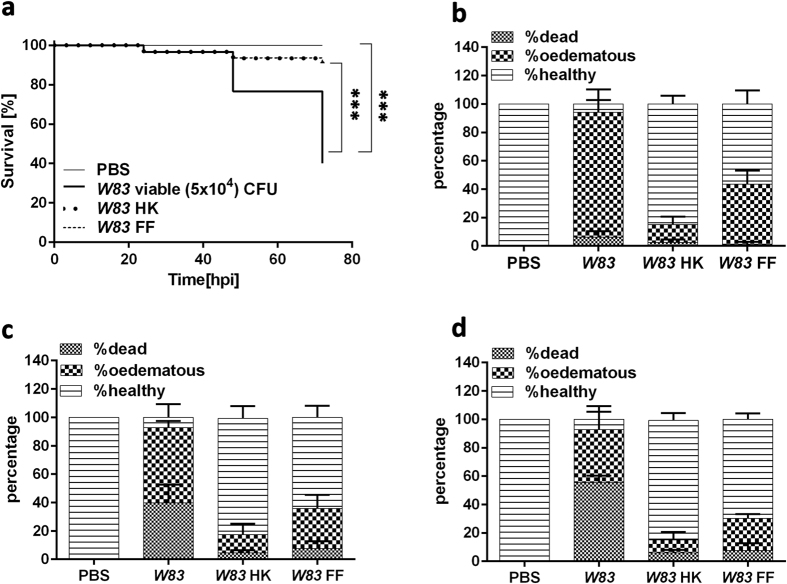
Heat and formalin treatment of *Pg W83* decreases its pathogenicity *in vivo*. Kaplan-Meier survival plots of zebrafish larvae infected with viable, heat-killed (HK) or formalin-fixed (FF) *Pg W83* (**a**). Percentage of dead, oedematous or healthy *Pg W83*-infected larvae after 24, 48 and 72 hpi respectively (**b**–**d**). Larvae were injected with viable, 60 °C HK or 10% FF Pg *W83* at 5 × 10^4 ^CFU. At least three individual experiments were performed (n ≥ 20 larvae per group in each experiment). ***p ≤ 0.001. Comparisons between survival curves were made using the log rank test. Differences between groups displaying oedema were measured using One-way ANOVA after normality was assured.

To determine if microbial viability was essential for virulence, zebrafish larvae were injected with viable, heat-killed (HK) or formalin-fixed (FF) *Pg W83* and mortality assessed. Kaplan-Meier analysis showed that HK or FF treatment of *Pg* markedly attenuated bacterial virulence causing a significant reduction (p < 0.0001) in zebrafish mortality after 72 hpi when compared to zebrafish injected with viable *Pg* (Fig. 5a). Although there was no difference in the percentage of zebrafish larvae killed with the different treatments after 24 h, more (88 ± 8%) zebrafish displayed oedemas compared to 42 ± 9% for FF and only 13 ± 5% for HK *Pg* (Fig. 5b). In zebrafish larvae injected with viable bacteria, almost all fish developed progressive disease symptoms that eventually led to the death of 56 ± 5% of larvae by 72 hpi (Fig. 5c,d). In contrast, the majority of the HK-infected embryos remained disease-free over a 72 h period (p ≤ 0.05, Fig. 5b–d) with only a minority experiencing oedema or mortality. A similar effect was observed for FF *Pg*-infected larvae although this group displayed consistently more oedema than the HK *Pg*-treated group over a 72 h period (Fig. 5b–d). As well as ensuring loss of viability heat treatment and fixation may also affect the activity of virulence factors, in particular those associated with enzyme activity. We found that treatment with elevated temperatures of 60 °C almost abolished the activity of both lysine (Kgp) and arginine (RgpA and RgpB) specific gingipains, whereas fixation of bacteria with 10% formalin reduced the activity of these gingipains by 60% (lysine specific) and 90% (arginine specific) respectively (Supplementary Fig. 2) indicating that these proteins may be important in mediating virulence.

### Gingipains are crucial virulence factors of *P. gingivalis* in zebrafish systemic disease

**Figure 6:**
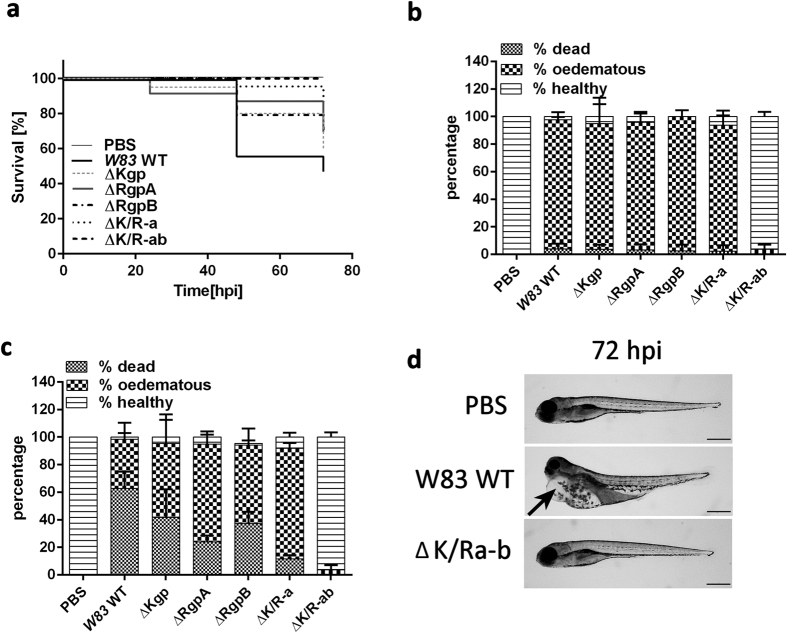
Gingipains are crucial virulence factors of *Pg* in the zebrafish larvae infection model. Kaplan-Meier survival plots of zebrafish larvae infected with *Pg W83* (WT), ΔRgpA deficient mutant, ΔRgpB deficient mutant, ΔKgp deficient mutant, Kgp and RgpA deficient mutant (ΔK/Ra) or gingipain null mutant (ΔK/Ra-b) (**a**). Percentage of dead, oedematous or healthy WT *Pg W83* or gingipain mutant-infected larvae after 24 (**b**) and 72 hpi (**c**) respectively. Morphology of zebrafish larvae 72 hpi with WT or ΔK/Ra-b mutant of *Pg W83* (**d**). PBS was injected as a control. All experiments were performed at least 3 times. Bars represent means ± SD. In all experiments WT *Pg* or its gingipain mutants were injected into 30 hpf zebrafish larvae at 5 × 10^4 ^CFU. Black arrow indicates oedemas in WT *Pg W83*-infected larvae. Scale bar = 500 μm. Comparisons between survival curves were made using the log rank test. Differences between groups displaying oedema were measured using One-way ANOVA after normality was assured.

To examine whether gingipains play a role systemic disease, zebrafish larvae were infected with wild-type (WT) parental strain *W83*, single (ΔKgp, ΔRgpA or ΔRgpB), double (ΔK/Ra) or triple (ΔK/Ra-b) gingipain-deficient mutants. Removal of any of the gingipains caused an increase in the survival rate of infected larvae compared to WT *W83* and this effect was additive with respect to the double or triple mutants whose mortality rates were similar to PBS controls (p ≤ 0.01 for ΔK/Ra and p ≤ 0.0001 for ΔK/Ra-b; Fig. 6a). With the exception of the ΔK/Ra-b triple mutant, zebrafish larvae injected with any of the mutated strains of *Pg* caused significant oedema in the first 24 hpi although there was little mortality (Fig. 6b). By 48 and 72 hpi (Fig. 6c) the levels of oedema remained the same; however, larvae mortality was increased to 60% for WT *W83 Pg* and 40% for lysine-deficient mutant (ΔKgp). Levels of larvae mortality were slightly, but not significantly, reduced upon infection with the arginine-deficient mutants ΔRgpA and ΔRgpB compared to ΔKgp. The ΔK/Ra-deficient mutant produced mortality levels of ~10% after 72 hpi whilst gingipain triple mutants (ΔK/Ra-b) were virtually avirulent, producing a larvae phenotype similar to PBS controls (Fig. 6c,d).

**Figure 7:**
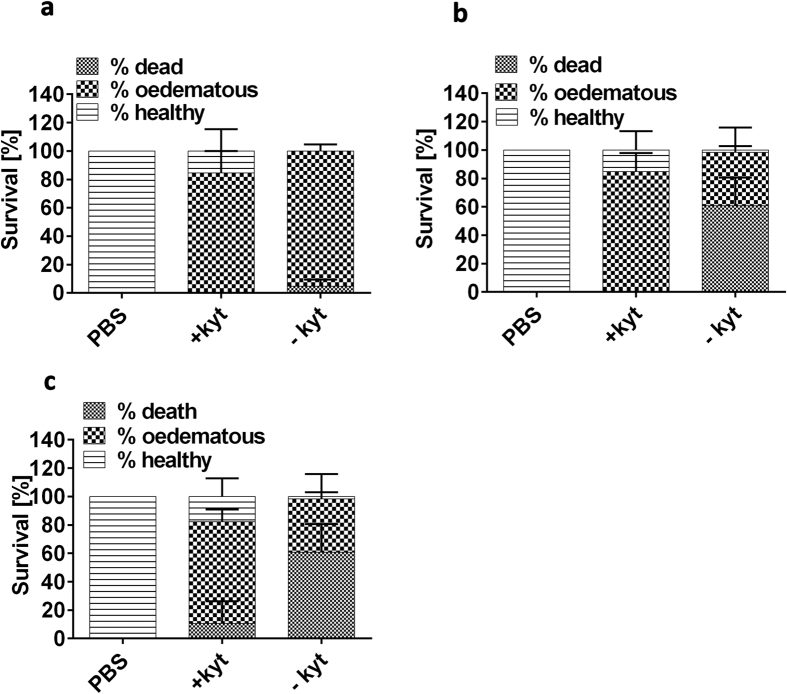
Effect of gingipain inhibitor KYT on zebrafish larvae mortality and morbidity. Percentage of dead, oedematous or healthy zebrafish larvae infected with 5 × 10^4 ^CFU *Pg W83* at 30 hpf in the absence or presence of KYT inhibitor after 24 (**a**), 48 (**b**) and 72 hpi (**c**) respectively. PBS was injected as a control. At least three individual experiments were performed and bars represent means ± SD. Differences between groups displaying oedema were measured using One-way ANOVA after normality was assured.

To confirm these findings we used a specific inhibitor of arginine and lysine gingipains (KYT)^24^ on *W83 Pg* to directly inhibit the proteolytic activity before systemic injection. The amount of inhibitor required to completely inhibit gingipain activity was determined *in vitro* before use *in vivo* (data not shown). We found that infection with *W83* + KYT significantly (p < 0.001) reduced larvae mortality by 48 and 72 hpi compared to those injected without inhibitor (Fig. 7a–c). However, the presence of gingipain inhibitor did not influence the number of larvae displaying signs of oedema compared to WT-infected *Pg* over a 72 h period (Fig. 7a–c).

## Discussion

There is increasing evidence to suggest that *Pg* influences the progression of chronic systemic conditions such as cardiovascular disease^25^,^26^. In support of this *Pg* has been found in atherosclerotic plaques^27^,^28^ and has been found to influence myocardial infarction in a murine model^29^. In this study we used zebrafish larvae, an *in vivo* model system that has been successfully used to investigate host-pathogen interactions^30^ to examine the effects of *Pg* on the circulatory system. We used a well-established systemic infection route^20^,^31^ along with genetically engineered zebrafish larvae that allowed us to examine, for the first time, the interaction of *Pg* with the vasculature at the cellular level *in vivo*.

We found that systemic infection with *W83*, a highly virulent strain of *Pg* compared to other strains tested, lead to dose-dependent disease severity and mortality in larvae^32^,^33^. A similar dose-dependent disease effect has been previously observed using a *Drosophila melanogaster* infection model albeit via a non-systemic route^34^. In addition, we observed extensive damage to the heart and surrounding tissue associated with pericardial oedemas. Microbial-mediated pericarditis in humans often leads to pericardial oedema and DNA from *Pg* along with that from other oral bacteria have been isolated from pericardial fluid and in endocarditis lesions^35^ suggesting that, although exaggerated, the disease process in zebrafish may reflect those in humans.

Using real-time *in vivo* confocal imaging we observed *Pg* both closely associated to and within endothelial cells of the vasculature, confirming *in vitro* data that *Pg* can adhere and be internalized by endothelial cells^36^. In addition, we found *Pg* deep within tissues showing that once internalized *Pg* are able to survive within endothelial cells and be released into tissues *in vivo*^37^,^38^. Li *et al*. showed *in vitro* that *Pg* can be transmitted intracellularly from cell to cell as a mechanism of spreading within the endothelium, and that these bacteria were able to replicate once within newly infected cells and then be released for further uptake^39^. Although we could recover viable *Pg* from infected zebrafish, we were are unable to determine if the bacterium replicated *in vivo* and so further studies are required to resolve this issue.

Although innate immune cells rapidly internalized and phagocytosed bacteria we also observed marked numbers of *Pg* in the circulatory system that was associated with significant damage and oedema in several tissues, most noticeably the pericardium and yolk sac. Moreover, this pathology was dependent upon the presence of gingipain proteases as lysine and arginine gingipain-deficient strains were both significantly less virulent, suggesting both types of gingipains can mediate tissue damage. Indeed, gingipain-null (ΔK/Ra-b)-infected zebrafish displayed no disease symptoms.

The importance of gingipains in causing periodontal as well as vascular damage in murine models has been previously highlighted^40^,^41^ and more recently gingipains were found to mediate myocardium damage in a murine model of *Pg*-aggravated myocardial infarction^29^. Several elegant studies have shown that either the lysine or arginine-specific gingipains mediate tissue damage by cleaving and therefore inactivating many important host extracellular matrix molecules including laminin, fibronectin and collagen, leading to loss of cell-matrix contacts that are crucial for tissue integrity and cell survival^42^. Moreover, this tissue destruction is exacerbated by *Pg*-mediated activation of matrix metalloproteinases, in particular MMP3^43^ and MMP9^44^. As well as disrupting cell-matrix interactions, there is *in vitro* evidence that gingipains also disturb endothelial cell-to-cell contacts. Sheets *et al*. found that lysine and arginine gingipains cleaved N- and VE-cadherin as well as integrin β1 expressed by monolayers of human microvascular endothelial cells leading to cell detachment along with caspase-mediated and caspase-independent apoptosis^45^,^46^. Given this data it is plausible that the increased vascular permeability leading to oedema we observed in systemically infected zebrafish may be due to gingipain-mediated damage of adhesion molecules leading to loss of endothelial adherin, gap and tight junction integrity. This may be further aggravated by reduced endothelial contacts with underlying connective tissue or support cells in more mature vessels. The model established here will pave the way for further studies to measure the effect of *Pg* and its virulence determinants on vascular leakage using a range of zebrafish lines open to the field.

Recently, small peptide analogs (KYT-1 and KYT-36) that are selective inhibitors of Kgp and Rgp, respectively^47^,^48^ have been developed. These inhibitors have been shown to suppress the pathogenic potential of *Pg* in murine models^24^. Similarly, we found that zebrafish systemically infected with WT *Pg W83* along with KYT1 and KYT36 displayed significantly reduced mortality and disease symptoms, suggesting not only that these inhibitory molecules show therapeutic promise but that the zebrafish infection model may be used to screen other therapeutic agents and strategies aimed at *Pg* or other bacterial-mediated systemic diseases.

In summary, this study reveals that zebrafish larvae represent a powerful novel vertebrate model system to examine the role of *Pg* and potentially other periodontal bacteria in cardiovascular disease or endocarditis. Additionally, we demonstrated for the first time that *Pg* gingipains are crucially important in mediating systemic disease. We believe that this *in vivo* model system will provide an amenable tool to further examine the mechanism of *Pg*-mediated systemic disease at the cellular and molecular level.

## Methods

### Bacteria, zebrafish strains and culture conditions

All strains of *Pg* and *Danio rerio* used in this study are listed in Table 1. Wild-type *Pg* W83 strain and its isogenic mutants were maintained on Schaedler (BTL Spolka, Poland) anaerobe blood agar (ABA) plates supplemented with 0.5 mg/L menadione sodium bisulfate, 0.25 g/L L-cysteine-HCl, 5 mg/L hemin, 5% (v/v) defibrinated horse blood and antibiotic (1 μg/ml tetracycline or 5 μg/ml erythromycin) when appropriate. For liquid cultures, strains were grown in hemin, menadione and L-cysteine supplemented Schaedler broth. ATCC33277 and FDC 381 strains were maintained on fastidious anaerobe agar (Lab M, Bury, UK) supplemented with 5% (v/v) oxylated horse blood (Oxoid, Basingstoke, UK). For growth in liquid cultures these strains were grown in brain-heart infusion broth (BHI; Oxoid) supplemented with 5 g/L yeast extract (Oxoid), 5 mg/L hemin and 0.5 mg/L menadione. Agar plates or liquid cultures were incubated at 37 °C in an anaerobic chamber (80% N_2_, 10% CO_2_ and 10% H_2_). For experiments, strains were grown in broth overnight at 37 °C in anaerobic conditions after which fresh cultures were set to an optical density (OD_600_) equal to 0.1 and cultured until late log phase. Bacteria were harvested by centrifugation at 6,000 × g for 10 min, washed with PBS and resuspended at 5 × 10^9 ^CFU/ml. For formalin fixation, *Pg* was resuspended in 10% PBS-buffered formalin and incubated overnight at 4 °C. For heat killing, bacteria were resuspended in PBS and incubated in a water bath at 60 °C for 30 min. The loss of bacterial viability was confirmed by culture on ABA plates. For inhibition of gingipain activity, *Pg* (OD_600_ = 1.0) were pretreated with KYT-1 or KYT-36 inhibitors^24^ at a final concentration of 2 μM and bacteria were incubated for 30 min at 37 °C in anaerobic conditions; after which, bacteria were washed once with PBS, resuspended at 5 × 10^9 ^CFU/ml and injected into zebrafish larvae. Zebrafish maintenance and experimental work was performed in accordance with UK Home Office regulations and UK Animals (Scientific Procedures) Act 1986. Ethical approval was given by the University of Sheffield Local Ethical Review Panel. London wild-type (LWT) inbred zebrafish larvae were obtained from The Bateson Centre, University of Sheffield. The transgenic *kdrl:memRFP* strain was used to enable visualization of the endothelium as previously described. All larvae were maintained in E3 medium at 28.5 or 30 °C according to standard protocols and monitored for up to 4 days post-fertilization (dfp).

### Microinjection of *P. gingivalis* onto zebrafish embryos

*Pg* were injected systemically by direct inoculation into the common cardinal vein (Duct of Cuvier) of dechorionated larvae at 30 hpf as previously described with PBS used as control^20^. Briefly, tricaine-anesthetized larvae were embedded and positioned in a solution of 3% (w/v) methylcellulose (Sigma Aldrich) in E3 medium on glass slides. Larvae were injected individually using micro-capillary needles loaded with known concentrations of *Pg* and were monitored for disease development and survival for up to 72 hpi unless otherwise stated. Zebrafish viability was assessed by examining the presence of a heart beat and blood flow within the circulation. Live imaging of anesthetized zebrafish larvae was performed using spinning disc confocal microscopy (Perkin Elmer). At least 20 zebrafish were injected per treatment group in all experiments.

### Evaluation of gingipain activity

To evaluate gingipain proteolytic activity *Pg* cultures were adjusted to OD_600_ 1.0. Ten microliters (for Rgp) and 20 μL (for Kgp) of bacterial suspension were pre-activated in TNCT activity buffer (50 mM Tris-HCl pH 7.5, 5 mM CaCl_2_, 150 mM NaCl, 0.05% Tween-20 supplemented with 10 mM L-cysteine-HCl neutralized with 10 mM NaOH) for 5 minutes at room temperature in a total volume of 200 μL in a 96-well plate. Substrate (0.4 μL, final concentration 200 mM) was added and the rate of its hydrolysis was recorded at 405 nm using a spectrophotometer as described previously^49^. N-(p-Tosyl)-Gly-Pro-Lys-*p*-nitroanilide and benzoyl-arginine-*p*-nitroanilide (BApNA) (both from Sigma Aldrich) were used as substrates specific for Rgps and Kgp, respectively.

### Histological processing of zebrafish

Infected and control zebrafish were fixed in 10% PBS-buffered formalin for at least 24 h and then paraffin wax-embedded. Tissue sections (5 μm) were cut, dewaxed in xylene, rehydrated through a series of alcohols to water and stained with haematoxylin and eosin (H&E). Sections of zebrafish were then dehydrated, mounted in DPX and images taken using an Olympus BX51 microscope and a IIIu camera with associated Cell^D software (Olympus, GmbH, Münster, Germany).

### Labeling of *P. gingivalis* and fluorescence microscopy

To visualize bacteria and to monitor phagocytosis *in vivo, Pg* were fluorescently labeled with fluorescein-5-EX and/or pHrodo red succinimidyl ester (Life Technologies, Paisley, UK) respectively. Prior to injection, a 200 μL bacterial suspension of *Pg* (in PBS; pH 9) was simultaneously mixed with 0.25 μL fluorescein-5-EX (10 mg/ml) and/or 0.25 μL of pHrodo red (1.7 mg/ml) for 30 min at 37 °C in the dark with shaking (100 rpm). Bacteria were washed twice with PBS (pH 8) to remove excess dye, resuspended in PBS (pH 7.4) to an OD_600_ 5.0 (~5 × 10^9 ^CFU/ml) and 2 nL injected into each zebrafish. At 2 hpi, six infected larvae were anesthetized, mounted in 1% low-melting-point agarose in E3 medium on coverslips. Fluorescent bacteria were visualized using either a spinning disc laser scanning confocal microscope (Perkin Elmer) and images acquired and processed using Volocity software (Improvision, Perkin Elmer) or a Zeiss Z1 light-sheet microscope with Zen Black 2014 software.

### Data and statistical analysis

All data presented is from at least 3 independent experiments with results expressed as the mean ± SD. Survival data were evaluated using the Kaplan-Meier method and comparisons between individual curves were made using the log rank test. Differences between groups displaying oedema were measured using One-way ANOVA after normality was assured. All analysis was performed using Graphpad Prism v6.0 (GraphPad, La Jolla, CA) and statistical significance was assumed if p < 0.05.

## Additional Information

**How to cite this article**: Widziolek, M. *et al*. Zebrafish as a new model to study effects of periodontal pathogens on cardiovascular diseases. *Sci. Rep.* **6**, 36023; doi: 10.1038/srep36023 (2016).

## Supplementary Material

Supplementary Information

Supplementary Information

## Figures and Tables

**Table 1:** *P. gingivalis*, zebrafish strains and bacterial culture conditions used in this study.

Strains	Genotype/Description	Reference
P. gingivalis		
W83	Wild-type	Reference strain^50^
ΔK/Ra-b	*kgp*^∆598-1732^::Tc^r^ *rgpA*^-^::Cm^r^ *rgpB*^∆410-507^::Em^r^	51
ΔRgpA	*rgpB*^ + ^::Em^r^ *rgpA*^-^::Cm^r^	52
ΔKgp	*kgp*^∆598-1732^::Tc^r^	53
ΔK/Ra	*kgp*^∆598-1732^::Tc^r^ *rgpA*^-^::Cm^r^	54
ΔRgpB	*rgpB*^∆410-507^::Em^r^	52
FDC 381	Wild-type	Forsyth Dental Center, Boston, MA
ATCC33277	Wild-type	55
Zebrafish		
Tg(*kdrl:memRFP*)		56
