# Supplementary material for: Zebrafish as a new model to study effects of periodontal pathogens on cardiovascular diseases
Source: Sci Rep. 2016 Oct 25;6:36023. doi: 10.1038/srep36023 (PMC5078774; doi:10.1038/srep36023)
Supplement: Supplementary Information [file srep36023-s1.pdf]

Zebrafish as a new model to study effects of periodontal pathogens on cardiovascular diseases

Magdalena Widziolek[1], Tomasz K. Prajsnar[2,3], Simon Tazzyman[4], Graham P. Stafford[5], Jan Potempa[1,6], and Craig Murdoch[5*]

Supplementary Figure 1

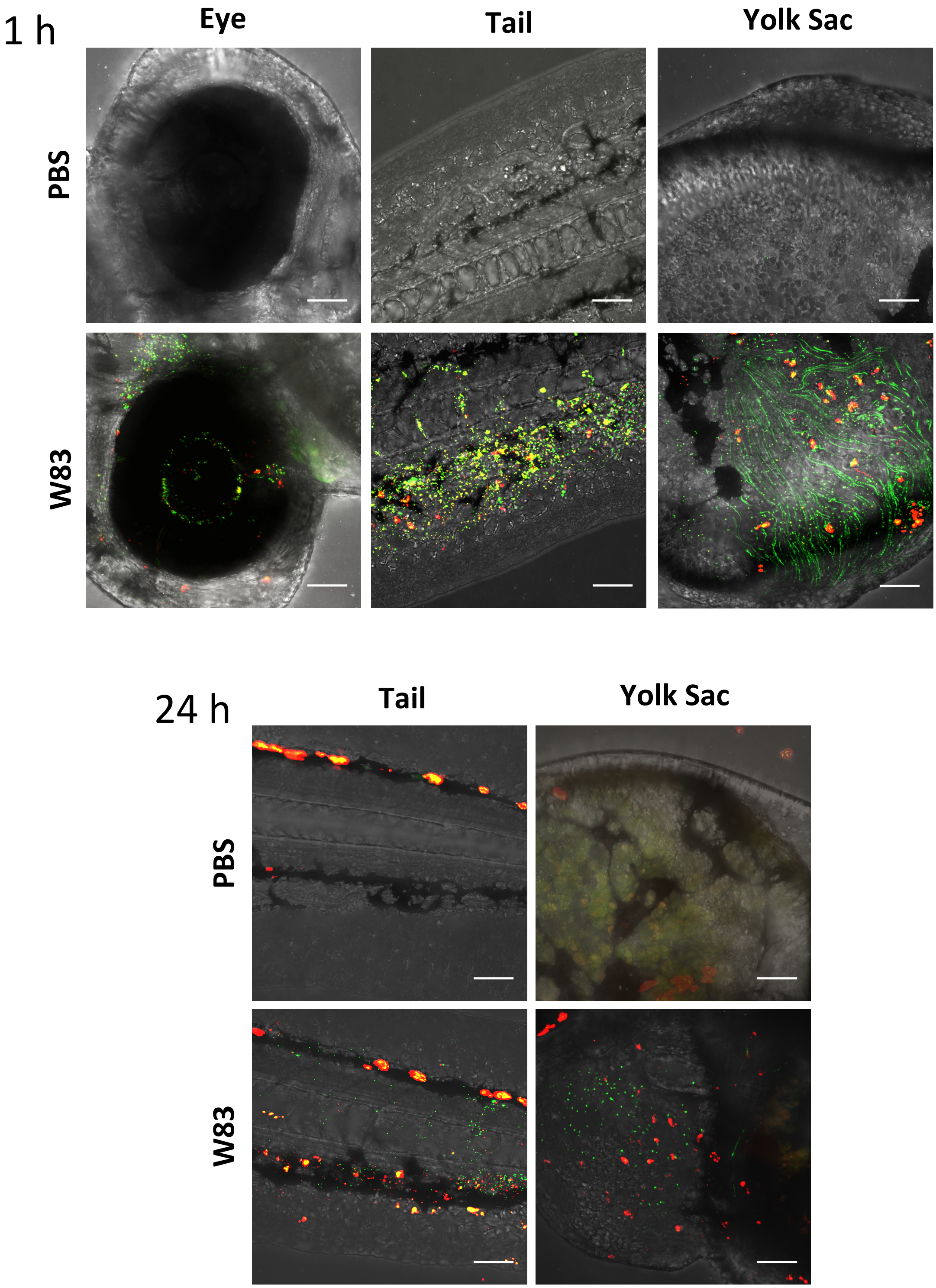

SF. 1. Confocal images of zebrafish larvae at 1 and 24 hpi following systemic infection with 5 x 10^4 CFU fluorescein and pHrodo labelled *Pg W83*. Small green dots represent non-phagocytosed *Pg* whilst small red dots are phagocytosed *Pg*. The large red staining in the PBS controls in the tail image is due to increased pigmentation along the back of the larvae at this stage or larvae development. Similar pigmentation was observed in the eye at 24 hpi masking the presence of *Pg* in this tissue and so this data is not shown. Images show the presence of less *Pg* in the tissues at 24 hpi with increased levels of phagocytosis. W83 Pg were injected into 30 hpf zebrafish larvae at 5 x 10^4 CFU in each experiment. Scale bar = 50µm.

Supplementary Fig. 2

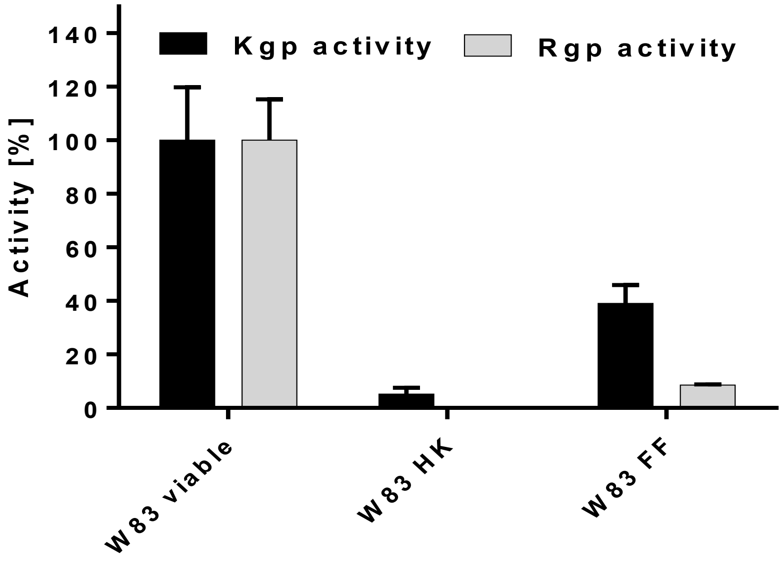

SF.2 Kgp and Rgp activity in cultures of viable, heat-killed (HK) and formalin-fixed (FF) wild-type *Pg* W83. The activity of HK or FF *Pg* W83 was calculated as the percentage of the activity of viable bacteria.

Supplementary Video: Light sheet real-time move of fluorescein-labelled *Pg* W83 adherent to the beating heat tissues of zebrafish larvae. *kdrl:memRFP* transgenic zebrafish larvae were infected with 5 x 10^4 CFU fluorescein-labelled *Pg W83* at 30 hpf. Small green dots represent *Pg* W83. Red = zebrafish endothelium.
